# Spectral Analysis of EEG in Familial Alzheimer's Disease with E280A Presenilin-1 Mutation Gene

**DOI:** 10.1155/2014/180741

**Published:** 2014-01-02

**Authors:** Rene Rodriguez, Francisco Lopera, Alfredo Alvarez, Yuriem Fernandez, Lidice Galan, Yakeel Quiroz, Maria Antonieta Bobes

**Affiliations:** ^1^Clinical Neurophysiology Department, Cuban Neuroscience Center, Havana, CP 10400, Cuba; ^2^Antioquia University, Medellin, CP 1226, Colombia; ^3^Cognitive Department, Cuban Neuroscience Center, Havana, CP 10400, Cuba

## Abstract

To evaluate the hypothesis that quantitative EEG (qEEG) analysis is susceptible to detect early functional changes in familial Alzheimer's disease (AD) preclinical stages. Three groups of subjects were selected from five extended families with hereditary AD: a Probable AD group (18 subjects), an asymptomatic carrier (ACr) group (21 subjects), with the mutation but without any clinical symptoms of dementia, and a normal group of 18 healthy subjects. In order to reveal significant differences in the spectral parameter, the Mahalanobis distance (*D* ^2^) was calculated between groups. To evaluate the diagnostic efficiency of this statistic *D* ^2^, the ROC models were used. The ROC curve was summarized by accuracy index and standard deviation. The *D* ^2^ using the parameters of the energy in the fast frequency bands shows accurate discrimination between normal and ACr groups (area ROC = 0.89) and between AD probable and ACr groups (area ROC = 0.91). This is more significant in temporal regions. Theses parameters could be affected before the onset of the disease, even when cognitive disturbance is not clinically evident. Spectral EEG parameter could be firstly used to evaluate subjects with E280A Presenilin-1 mutation without impairment in cognitive function.

## 1. Introduction

Alzheimer's disease (AD) is a neurodegenerative disorder in the elderly characterized by progressive dementia [1, 2]. The disorder probably begins many years before the first clinical symptoms are evident [3, 4]. Recent studies have demonstrated that during the presymptomatic phase, neuronal degeneration occurs even without the presence of clinical symptoms [5]. These make preclinical discrimination between people who will and will not ultimately develop AD critical for early treatment of the disease [6].

Neuropathological hallmarks of AD include macroscopic change as reduced brain weight with cortical atrophy and ventricular enlargements primarily due to neuronal loss in the temporal and parietal structures [7]. At the microscopic level, it can be found neurofibrillary tangles (intracellular aggregations of tau protein filaments) and amyloid plaques (extracellular aggregates of amyloid beta-peptides) that are particularly concentrated in the hippocampus, entorhinal cortex, and postcentral parietal neocortex. [2, 8, 9].

Recent advances in molecular genetics have allowed identifying individuals carrying defective genes predisposed to develop AD [10]. When the disease penetrance is high, the examination of apparently asymptomatic subjects carriers of defective genes allows early evaluation of different physiopathology's processes [11]. Up to now, three genes have been unequivocally related to familial forms of AD, the Presenilin-1 (PS-1) gene, accounting for 15–50% of the cases, the amyloid precursor protein (APP), and the Presenilin-2 (PS-2) mutation which have been identified in less than 1% [10].

In a genetic analysis, the findings, of mutations that produce an autosomal dominant form of AD, in a patient with dementia or in a family carrying dominant autosomic form of the disease, allow a diagnosis with nearly 100% of certainty [1]. While the cause(s) of the most common AD the late-onset form is unknown, mutations in Presenilin-1 cause many cases of familial the early onset AD [13].

In a community based at Antioquia, Colombia, there is a well-documented form of early onset familial AD related with glutamic acid to alanina mutation at the codón 280 of chromosome 14, in the Presenilin-1 gene [12]. This mutation provides autosomal dominance inheritance, with virtually 100 % penetrance. Clinically, this phenotype cannot be distinguished from sporadic AD except for the early aged onset. The patients had a mean age at onset of 46.8 years [12]. A clinical diagnosis accuracy of approximately 85% of detection rate is commonly achieved, by a procedure of exclusion after structural or functional imaging tests including quantitative electroencephalography, laboratory, and psychometric test [13]. Annual conversion rate, from normality to dementia of AD type, it ranges between 0.2% and 4% whereas, from mild cognitive impairment to AD, is between 6% and 25%. It is an open issue with important clinical implication whether or not mild cognitive impairment is essentially prodromal stage of AD [14–16].

In the aging brain—including the AD ones during pre-clinical conditions—plastic compensatory remodeling guarantees functional maintenance, so that the neuronal and synaptic death can occur in the absence of symptoms for an unknown period of time that might last years or even decades. This mechanism of “cognitive or brain reserve” motivates the use of instrumental markers of AD in association with standard assessment of cognitive functions [7]. Few studies have assessed EEG measures over the course of dementia progression.

In our sample, there are groups of asymptomatic carrier which are going to develop the AD with 100% of accuracy because his mutation in the Presenilin-1 gene provides autosomal dominance inheritance with virtually 100% of penetrance. Systematic studies of this familial AD have enabled the identification of subjects that carry the mutation but without clinical signs or symptoms [12]. This condition allows finding individuals at preclinical stages of AD, permitting the early electrophysiological evaluation with the quantitative EEG measure.

Multiple techniques have been used to evaluate the pathophysiological processes underlying early stages of AD. Among them, the quantitative analysis of digital electroencephalogram (EEG) has been introduced as a nonexpensive, noninvasive, and objective tool for evaluating dementia. Longitudinal analyses of brain EEG rhythms have provided objective evidences of disease progression from MCI to AD [17, 18]. Previous studies using the EEG in demented patients have reported high sensitivity in detecting a diffuse organic damage, but low specificity in determining an etiologic diagnosis [19]. In the last years, several research groups have started investigating the potentiality of electroencephalogram for diagnosis AD. However, in our knowledge, there are no previous research using EEG signal for the diagnosis of AD subjects to ensure 100% who develop the disease in the future will develop the disease.

Many researches have shown that mild cognitive impairment and AD cause EEG signals to slow down and mild cognitive impairment and AD are associated with increase of power in slow frequencies (delta- and theta-band) and a decrease of power in fast frequencies (alpha- and beta-band). Nevertheless, increased gamma-band power has been reported in mild cognitive impairment and AD patients compared to healthy age matched control subjects [20].

The most often electroencephalographic findings in AD patients are (a) severe slowing of background activity with an increased power in slow EEG activity [21–26] and (b) a concomitant decrease of the power at fast (alpha and beta) EEG frequency ranges [21, 27, 28]. Many researches also hypothesize that the earliest modifications of the EEG occur in the beta- and theta-bands, while changes in alpha and delta bandwidths appear later in the time course of the disease [2, 29]. However this pattern is not universally found [29, 30]. Several studies have shown a close relationship between an increase in the slow frequency and the degree of cognitive impairment in these patients [26, 31–35]. The quantitative EEG has been also used to evaluate the treatment response with cholinesterase inhibitors [36] and the dementia follow-up [28, 37].

When compared to healthy normal elderly subjects, AD patients evidence high power for delta and theta and low power for posterior alpha (8–12 Hz) and/or beta (13–30 Hz) frequencies [2, 7, 38–42]. Some of these EEG changes could differentiate dementia diagnosis, as the strong decline of posterior slow frequency alpha sources that occurs specifically for mild AD group when compared to vascular dementia and normal elderly groups. In addition, abnormal wide theta sources characterized cerebrovascular dementia patients [41]. EEG abnormalities were associated with altered regional blood flow/metabolism and impaired global function as evaluated by minimental state examination (MMSE) [2, 33, 43].

Nevertheless, in the earliest stages of AD, electroencephalographic patterns have not been completely characterized. Ambiguous results have been reported; Nobili et al. [31] found no EEG alteration or worsening in 50% of early AD cases in a one-year follow-up study. Other authors described a delay in the peak of the dominant frequency [33, 44]. Different recording methods and analysis procedures have been used. Normally, the classical frequency band analysis has a poor resolution and may overlook slight, but important, changes in the spectra [33, 45]. This drawback may be solved by the use of narrower bands.

Of note, early stages of AD (even preclinical) are typically associated with slowing down resting occipital alpha rhythms, namely, a decrease of the individual alpha frequency (IAF) peak in power density [46]. The IAF peak, defined as the frequency associated with the strongest EEG power at the extended alpha range [47], should be always taken into account in EEG studies in AD subjects, since power changes in theta and alpha bands might be dependent phenomena. Furthermore, the conventional partition of EEG power into many conventional frequency bands allows the comparison of the results with those of most of the field studies but may prevent the separation of independent EEG rhythms or sources [48].

The aim of the present study is to evaluate the hypothesis that quantitative EEG analysis is able to detect early functional changes in preclinical stages of familial AD in ACr and clinically normal subjects. We believe that this research may help to identify an electroencephalographic pattern that could distinguish which genetically predisposed subjects will develop more rapidly the disease which in turn may in the long term improve the reliability of EEG as a diagnostic tool for AD.

## 2. Methods

### 2.1. Subjects

Three groups of subjects were selected from five extended families affected by early onset AD due to an E280A Presenilin-1 mutation [13]. These were as follows.

- A probable AD group: eighteen patients with the E280A Presenilin-1 mutation are diagnosed as “probable AD”, but still with mild symptoms of the disease according to the minimental state examination [49] (MMSE: 15–23, and the functional assessment stages (FAST <5).
- An ACr (asymptomatic carriers) group there are twenty-one subjects, with the mutation but without any clinical symptoms of dementia (MMSE: >23 and FAST = 1).
- A normal group there are eighteen healthy subjects without history of neurological or mental disease, not carrying the mutation (MMSE: >23 and FAST = 1). The subjects of this group were selected from the families in which there are any members with E280A Presenilin-1 mutation with probable AD or ACr.

The exclusion criteria were severe physical illness, psychiatric or neurological disorders associated with potential cognitive dysfunction, and other dementia conditions (fronto-temporal dementia, dementia associated with Parkinsonism, Lewy body disease, pure vascular or prion dementia, etc.). Subjects with alcohol/drugs abuse, regular use of neuroleptics, antidepressants with anticholinergic action were also excluded.

Informed consent for participation was obtained from all subjects according to a general protocol approved by the Human Subjects Committee of University of Antioquia, Medellin, Colombia.

The presence of signs or symptoms of AD was assessed using the criteria outlined by the National Institute of Neurological and Communicative Disorders and the Alzheimer's disease and Related Disorders Association (NINCDS-ADRDA) [50] and the DSM-IV criteria.  Table 1 shows the mean values of demographic and clinical characteristic of the probable AD, ACr and Normal groups as well as the results of a one-way ANOVA for each of the variables.

### 2.2. EEG Recordings

EEG recordings were obtained from subjects comfortably resting with their eyes closed. Subjects were continuously monitored in order to detect drowsiness. EEG data were recorded from 19 electrodes positioned according to the 10–20 international system. The ground electrode was placed in Fpz. The short-circuited left and right mastoid served as reference for all 19 channels. The recordings were used offline to rereference to common average. Electrode impedance was kept below 5 Kohms. EEGs were recorded with the FENIX System (NEURONIC S.A), they were amplified with a gain of 512, a filtering band pass of 0.5–30 Hz, and a sampling rate of 200 Hz. A 60 Hz notch filter was also used. The EEGs were visually inspected offline.

### 2.3. EEG Analysis

For quantitative analysis, 24 artefact-free epochs of 2.56 seconds duration were selected. The fast Fourier transform was computed for each segment. Broad band spectral parameters (absolute power, relative power, and mean frequency) were calculated in four electroencephalographic classic bands: delta (0.5–3.5 Hz), theta (3.5–7.5 Hz), alpha (7.5–12.5 Hz), and beta (12.5–19.1 Hz) while narrowband frequency model was computed with a frequency resolution of 0.39 Hz from 0.78 to 19.14 Hz [51–53]. A logarithmic transformation was applied to the spectral estimates to obtain an approximate Gaussian distribution. The spectral power at each electrode was normalized to the spectral power averaged across all frequencies (0.5–19 Hz) and electrodes.

The individual alpha frequency (IAF), as an anchor frequency, was selected according to literature [46]. The IAF is defined as the frequency associated with the stronger EEG power at the extended alpha range. The frequencies bands were adjusted individually for each subject, by using IAF as the cut-off point between the lower and upper alpha band.

### 2.4. Statistical Analysis

In order to reveal difference between groups the following steps were carried.

(I) The *z* standardized statistic was calculated for all spectral parameters

(1) $$\begin{array}{c} z=\frac{x-\mu \left(\text{age},\text{MMSE}\right)}{\sigma \left(\text{age},\text{MMSE}\right)}, \end{array}$$

where *μ* and *σ* are the mean and standard deviation (SD) estimated in the normal group. The *x* value is the observation of each subject. As EEG power in the theta and alpha frequency range has been described that is related to cognition and memory [47, 54]. MMSE and age were included as covariate for *Z* calculation. This was also supported by our results where we found a significance difference between mean values in each group in these covariates (Table 1). The mean and standard deviation values were computed using the regression functions obtained from the normal group using the covariates aged and MMSE with crossvalidation technique (leave one out) to compare a single individual to a population of “normal” individuals. In order to identify the measures that are deviant from normal and the magnitude of deviation, the *Z* score was computed for all variables based on his/her respective age, MMSE matched mean, and SD in the normal group.

(II) The components of *Z* vector tend to be highly correlated. For example, parameters from left-right homologous derivations tend to be symmetrical, the deviation of *Z* vector from normal group. This was carried out by consideration of the correlations between its components by means of Mahalanobis distance (*D* ^2^). These represent the direction of maximum deviation [55]. The formal definition is

(2) $$\begin{array}{c} D^{2}=Z^{T}\Sigma^{-1}Z, \end{array}$$

where *Z* is the vector according to step (I) and Σ^−1^ is the inverse covariance matrix of the *Z* vector. Following standard math notation *Z* ^*T*^ is the transpose of vector *Z*.

The application of *D* ^2^ for broad band frequency has been considered by John et al. in 1987 [56]. The *D* ^2^ is useful to combine deviations from the normal pattern in different spectral feature enhancing slight deviation at different frequency bands. This combination is in agreement with usual practice in mapping studies which involves the subjective analysis of deviation from the norm in multiple spectral maps.

The *D* ^2^ distance was calculated by selecting different parameters of the *Z* vector as follows:

1. taking in account all the parameters (here called global *D* ^2^).
2. considering all frequencies in a fixed region (five regions were considered: frontal (F3, F4, F7, F8, Fz), central (C3, C4, Cz), temporal (T3, T4, T5, T6), parietal (P3, P4, Pz), and occipital regions (O1, O2). (this was referred to as regional *D* ^2^),
3. considering all regions in a fixed frequency interval. Firstly, the classical broad band model was considered (delta, theta, alpha, and beta bands), and in a second place, two news bands were defined slow and fast band. The slow band contained frequencies from 0.5 to 7.5 Hz and the fast band frequencies from 7.5 to 19.14 Hz (here refered to as frequency *D* ^2^).

(III) To evaluate the diagnostic efficiency of *D* ^2^, receiving Operate Curves (ROC) models were used. The ROC curve was summarized by its accuracy index and SD, respectively. This is a measure of the probability to perceive abnormality between two groups. High ROC area values reflect higher accuracy.

The *P* value observed under null hypothesis was corrected by Bonferroni with *α* adjusted of 0.0010.

## 3. Results

**Figure 1:**
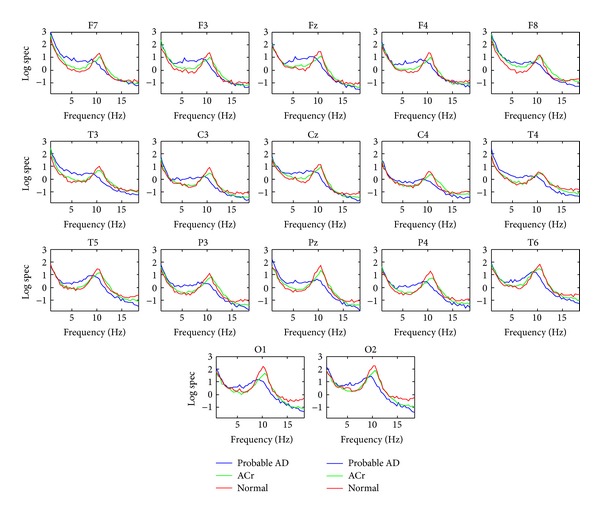
Averaged spectral power in the three groups. In *y*-axis are represented the values of the logarithm of the spectrum for each value of frequencies and derivation.

The average spectral logarithm was obtained for each group.  Figure 1 shows superimposed averaged log spectral power for each group of the narrow band model. In temporal regions, the probable AD group shows a higher increase of the power in the theta band compared to the ACr and the normal groups. On the other hand, the probable AD group showed a decrease of the alpha power with respect to the other two groups. The SD of the spectra for all frequencies and derivations in the groups were normal group 0.55, ACr group 0.87, and probable AD 1.02. The difference between these log spectral value should be demonstrated statistically, as a significant difference between the accuracy of classified using the individually distance to normal group (according to step (II) in section statistical analysis).

### 3.1. Global *D* ^2^

**Figure 2:**
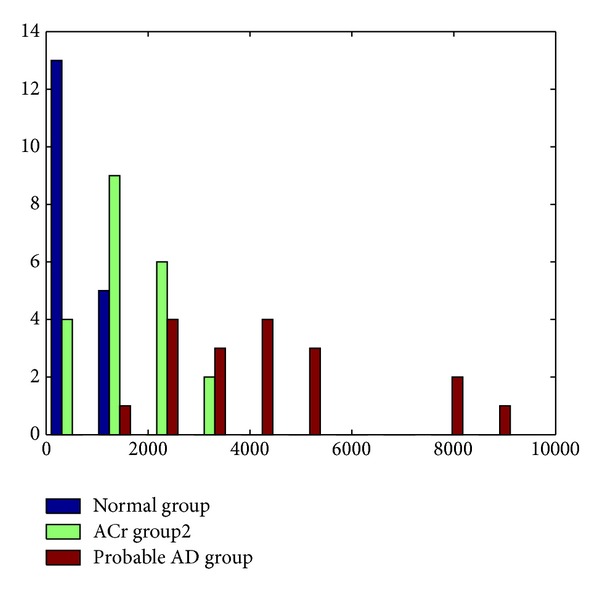
Histograms of *D* ^2^ calculated for the *Z* log spectra of the narrow band model. The *x*-axis shows the values of the Mahalanobis distance for each subjects. *Y*-axis shows the observed frequencies (number of subjects).

The *D* ^2^ was computed using the *Z* log spectra of the narrow band model. The histogram of the *D* ^2^ (Figure 2) shows that the maximal distance (in decrease order of magnitude) was reached in the Probable AD followed by the ACr group and the normal group.

The discriminative accuracy of *D* ^2^ was quantitative measured *q* by means of the ROC area. The areas were estimated between normal and ACr groups (area ROC = 0.90), normal probable AD groups (Area ROC = 0.98), and ACr-probable AD groups (area ROC = 0.92). That means that it is possible to separate ACr and probable AD groups from the normal group, but also that the diagnostic performance is higher to separate ACr and probable AD groups.

### 3.2. Regional *D* ^2^

In order to determinate the accuracy according to the regions and frequency bands,*D* ^2^ was calculated for selected component of the *Z* log spectra in five different regions: frontal (F3, F4, F7, F8, Fz), central (C3, C4, Cz), temporal (T3, T4, T5, T6), parietal (P3, P4, Pz), and occipital (O1, O2).

**Figure 3:**
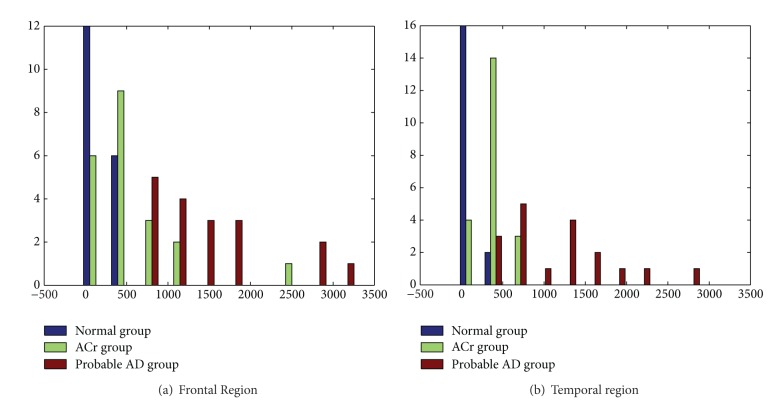
Histograms of *D* ^2^ for *Z* log spectral values in two regions of the three groups. In *x*-axis are represent the values of Mahalanobis distance for each subject. *y*-axis represents the observed frequencies.

ROC areas were obtained for five regions (see Table 2).  Figure 3 shows *D* ^2^ the histogram obtained for temporal and frontal regions. The maximal difference among the normal group and the other groups (in decrease order of magnitude) was localized in temporal, frontal, central, occipital and parietal regions. In the other hand, the best diagnostic performance between ACr and probable AD groups was obtained at the temporal region (area ROC = 0.94).

### 3.3. Frequency *D* ^2^

#### 3.3.1. Classic Bands

The *D* ^2^ applied to the broad-band model considered (delta, theta, alpha, and beta bands). The ROC areas were also obtained for each band of the broadband model (see Table 2).

The discrimination index using *D* ^2^ showed that beta band was more accurate to discriminate between normal and ACr groups (area ROC = 0.89) and between probable AD and normal groups (Area ROC = 0.99) than the rest of the classical bands.

#### 3.3.2. Slow and Fast Bands

**Figure 4:**
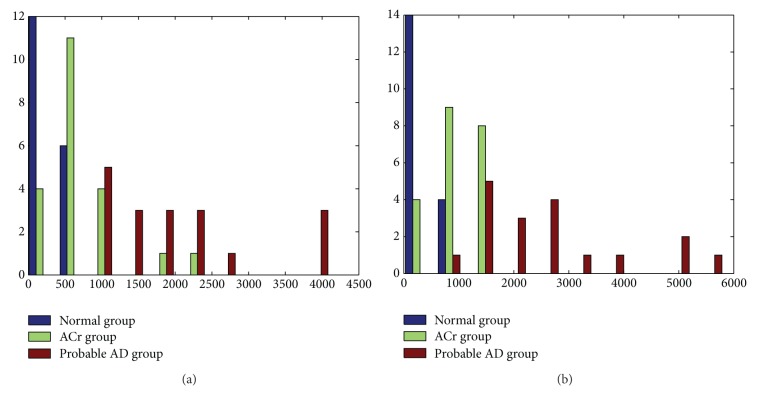
Histograms of *D* ^2^ calculated in (a) slow (delta theta) and (b) fast (alpha beta) frequencies for all regions of the three groups. The *x*-axis shows the values of the Mahalanobis distance for each subjects. *Y*-axis shows the observed frequencies (number of subjects).

Figure 4 shows the *D* ^2^ histogram for the slow and fast bands. The accuracy of classification in the fast band was higher than that in the slow frequency bands. In decrease order of magnitude, the best indexes were among normal probable AD groups (area ROC = 0.98), ACr probable AD groups (area ROC = 0.91), and normal ACr groups (area ROC = 0.89) (see Table 2).

## 4. Discussion

The goal of the present study was to determine the possible impact of spectral EEG analysis to detect early functional changes in preclinical stages of familial AD. The main finding of the present research was the presence of beta-bands alteration in ACr groups in the absence of clinical sign. Fast frequency bands change mainly in gamma-frequencies, in people with clinical sign of mild cognitive impairment. Other researches [57] also found a significant decrease of EEG power in the 14–18 Hz and 18–22 Hz in AD patients. Reduction of beta band power was correlated with severe cognitive dysfunction. The researcher suggested that a reduction of beta power is not only due to ageing, but may reflect an alteration of AD especially in the early stage, [58] This change in the EEG could be found in another dementia, but generally the subjects have clinical sign of cognitive disorders. However, the findings of difference in beta band activity in ACr when compare with normal group suggest the possibility that this disturbance in the cholinergic system begins before cognitive impairment appears. This finding could imply that beta band could be affected before the alteration in the gamma described in subjects with mild cognitive impairment. It is possible that early modifications in beta band as found in this study are not sufficient to produce clinical cognitive impairment but could be an early sign to develop the disorder in the future. The modification in delta and alpha bands observed in this study was similar to that previous reports in subjects with AD [59–61].

The importance of high frequency for cognitive process has been recently stressed in several studies [62–64]. Claus et al. [64] reported that loss of beta band power is an independent predictor of unfavorable prognosis in AD. Stam et al. [65] suggested that loss of beta band power may also be important for early diagnosis of AD.

Besides a corticocortical uncoupling progression, a decrease of synaptic coupling is likely to contribute selectively to reducing EEG coherence for faster rhythms, as observed in healthy humans by transient use of a cholinergic synaptic blocker like scopolamine [66]. Animal models suggest that acetylcholine reduction produces a decrease of high frequency EEG coupling and an increase of slow frequency coupling [67]. Significant drop in EEG synchronization in faster rhythms has also been correlated with decreased MMSE scores in MCI and AD patients [68]. Our findings suggest a decrease of functional connectivity in beta band in ACr. This may be explained by a loss of intracortical connections, which are essential for interactions between brain regions. These connections are known to be affected in AD.

On the other hand, our research showed changes in the EEG spectral parameters in frontal and temporal region likely the most early and sensible than other regions. A related study further supports the role of EEG as a noninvasive tool used in the early identification of dementia demonstrated the earliest subcortical and cortical changes, associated with neural decline [67] According to Başar and Güntekin [69], the left fronto parietal connections are highly affected by AD pathology primarily occurring within the parietal regions during the early stages of the disease.

In summary, our results suggest that the modification in the qEEG of subjects with genetic predisposition to develop AD is characterized by change in beta frequency band and modification in fronto-temporal regions of the spectral parameters before clinical sign of cognitive impairment appears in subjects with E280A Presenilin-1 mutation but this finding needs to be finding with increase of the number of subjects with this mutation and corroborates in another genetic form of dementia.

These results should be carefully handled because it has been reported that families with PS-1 mutation present a more severe clinic syndrome than families with PS-2 mutation or another genetic form of dementia. Nevertheless, these results open a possibility to recognize an electroencephalographic pattern that could distinguish which genetically predisposed subjects will develop more rapidly the disease and perhaps which subjects with mild cognitive impairment or sporadic AD form could suffer a quick deterioration in their cognitive function through functional disturbances that are indirectly present in the EEG activity. Another important issue is to quantify the severity of the disease using spectral EEG analysis to provide patients, ACr subjects, and their families with a more reliable prediction of the disease's course. An appropriate clinical treatment, even at early stages, begins with several actions by means of cognitive rehabilitation and planning for necessary social resources.

However, many crucial issues will need to be addressed before using EEG in the clinical practice as a tool for diagnosing AD. This research field offers sufficient opportunities for exciting and clinically relevant research and opens the possibilities to increase the number of subjects with this or another genetic form of dementia.

## Figures and Tables

**Table 1:** Demographic and neuropsychological data of interest of normal, ACr, and probable AD subjects.

	Normal	ACr	Probable AD	Anova
*N*	18	21	18	
Age (years)	41.8 (±7.5 SE)	39.9 (±7.30 SE)	49 (±5.03 SE)	F = 9.46, df = 2.54 ***P* < 0.0002**
Gender (F/M)	14/4	14/7	9/9	
MMSE	28.8 (±1.23 SE)	28.2 (±1.84 SE)	21.1 (±2.44 SE)	F = 67.52, df = 2.54 ***P* < 0.0000**
Education (years)	6.33 (±2.95 SE)	7.28 (±4.14 SE)	6.22 (±4.02 SE)	F = 0.47, df = 2.54 *P* < 0.62
IAF	9.36 (±0.63 SE)	9.41 (±0.9 SE)	8.72 (±0.9 SE)	F = 8.51, df = 2.54 ***P* < 0.0006**

MMSE: mini-mental scale examination; IAF: individual alpha frequency; SE: standard error; F: female; M: male; df: degree of freedom.

**Table 2:** The ROC areas and standard deviation for the different estimated *D* ^2^.

	Groups	ROC values	SD
Global *D* ^2^	Normal ACr	0.90	0.054
	Normal probable AD	0.98	0.024
	ACr probable AD	0.92	0.048

Regional *D* ^2^			
Frontal	Normal ACr	0.85	0.065
	Normal probable AD	0.98	0.024
	ACr probable AD	0.91	0.050
Central	Normal ACr	0.84	0.067
	Normal probable AD	0.98	0.024
	ACr probable AD	0.90	0.053
Temporal	Normal ACr	0.89	0.056
	Normal probable AD	0.98	0.024
	ACr probable AD	0.94	0.041
Parietal	Normal ACr	0.68	0.089
	Normal probable AD	0.98	0.024
	ACr probable AD	0.67	0.088
Occipital	Normal ACr	0.82	0.071
	Normal probable AD	0.98	0.024
	ACr probable AD	0.84	0.066

Bands *D* ^2^ (Classic bands)			
Delta-band	Normal ACr	0.77	0.079
	Normal probable AD	0.98	0.024
	ACr probable AD	0.88	0.058
Theta-band	Normal ACr	0.86	0.063
	Normal probable AD	0.98	0.024
	ACr probable AD	0.90	0.053
Alpha-band	Normal ACr	0.86	0.063
	Normal probable AD	0.98	0.024
	ACr probable AD	0.85	0.064
Beta-band	Normal ACr	0.89	0.056
	Normal probable AD	0.99	0.017
	ACr probable	0.98	0.024

Bands *D* ^2^ (slow and fast bands)			
Slow bands	Normal ACr	0.84	0.067
	Normal probable AD	0.98	0.024
	ACr probable AD	0.90	0.053
Fast bands	Normal ACr	0.89	0.056
	Normal probable AD	0.98	0.024
	ACr probable AD	0.91	0.050
